# Enzyme-Treated Soybean Meal Serves as an Effective Alternative to Fishmeal in the Diet of the Shrimp *Penaeus vannamei*

**DOI:** 10.1155/anu/2312302

**Published:** 2025-03-24

**Authors:** Jianchun Shao, Qingyin Zheng, Zhengbang Chen, Wenbo Zhu, Qiulei Ren, Kai Yuan, Linwei Yang

**Affiliations:** ^1^State Key Laboratory of Mariculture Breeding, Key Laboratory of Marine Biotechnology of Fujian Province, College of Marine Sciences, Fujian Agriculture and Forestry University, Fuzhou, Fujian, China; ^2^Fuzhou Haima Feed Co., Ltd., Fuzhou, Fujian, China; ^3^School of Life Science, Huizhou University, Huizhou, Guangdong, China

**Keywords:** enzyme-treated soybean meal, fishmeal, *Penaeus vannamei*, replacement

## Abstract

Research on finding alternative protein sources to replace fishmeal (FM) has become a central issue in the nutrition field. Extensive research has been carried out on the replacement of FM with soybean meal (SBM); however, little is known about the replacement of FM with enzyme-treated SBM (ESBM). In this study, five isolipidic and isonitrogenous diets were formulated by substituting FM with ESBM at the levels of 0% (FM, control diet), 5% (ESBM25), 10% (ESBM50), and 15% (ESBM75) which were fed to juvenile shrimp for 8 weeks. And we found that replacing FM with ESBM at 5%–10% levels in shrimp diets had no impact on shrimp growth performance and feed utilization. However, substituting 10% ESBM for FM in the shrimp diets promoted the expression of growth-related genes and maintained consistent intestinal microbiota compared to the control group. Replacing FM with 15% ESBM in the shrimp diets inhibited shrimp growth, suppressed mTOR gene expression, and promoted the proliferation of harmful intestinal bacteria. Furthermore, replacing FM with different ESBM did not affect the intestinal health of shrimp. Taken together, our research provides that replacing FM with 10% ESBM is feasible. These findings not only enrich our knowledge of FM proteinogen replacement but also provide a reference for the use of ESBM as a substitute for FM in commercial feeds for shrimp *Penaeus vannamei* as well as other shrimp species.

## 1. Introduction

Pacific white shrimp *Penaeus vannamei* is a significant economic species in global aquaculture. However, with the continuous expansion of intensive aquaculture, the demand for shrimp feed has surged. Fishmeal (FM) stands out as the paramount ingredient in aquaculture feed, which has become the primary protein source of aquaculture feed due to its high protein content, minimal antinutritional factors (ANFs), diverse amino acid composition, and high digestibility [1]. FM is in short supply and has soaring prices due to the overfishing of marine fish and climatic factors such as El Niño. Therefore, there is an urgent need to find alternative sources of protein for FM [2].

Plant protein sources are currently one of the main alternative sources of FM [3]. Soybean meal (SBM) is one of the most used plant protein sources in the alternative sources of FM due to its high protein content, cheap price, and abundant resources [4]. However, the presence of ANFs such as trypsin-inhibiting factors in SBM limits its extensive use in feed [5, 6]. It has been found that the nutritional shortcomings of SBM can be effectively improved by enzyme-treated SBM (ESBM), by increasing the content of low molecular weight proteins and reducing ANFs [7]. Partial replacement of FM with ESBM in the diet was found to effectively improve the intestinal health and antioxidant capacity of piglets while maintaining their growth performance unaffected [7]. Replacing FM with 2% ESBM in the diet improved the broilers' intestinal microbiota and growth performance [8, 9]. In aquaculture, replacing FM with 75% ESBM does not affect the growth performance and immunity of abalone [10]. However, the ESBM group, which contained fewer ANFs, exhibited a reduced abundance of intestinal microbiota, including *Halomonas*, *Zoobellella*, and *Bacillus* [10]. This reduction may be attributed to the lower levels of ANFs, which needs further experiments to be confirmed. In largemouth bass, replacing FM with 30% fermented SBM (FSBM) significantly increased the abundance of *Cetobacterium* and *Mycoplasma* of the intestine, which may be associated with the reduced levels of ANFs [11]. In Nile tilapia, the fermentation of plant-based feeds with *Lactobacillus acidophilus*, which contained fewer ANFs, improved the beneficial bacteria and reduced pathogenic strains in the intestine [12]. It is evident that partial replacement of FM with ESBM holds significant application value in piglets, broilers, abalone, largemouth bass, and Nile tilapia. However, further research is needed to assess the viability of partially replacing FM with ESBM for shrimps.

Growth performance, oxidative capacity, intestinal health, and microflora of aquaculture animals are important indicators used to evaluate other protein sources as alternatives to FM [5, 6, 13, 14]. Growth performance is the most direct indicator for evaluating the quality of aquaculture feeds, and oxidative capacity is an important indicator for maintaining the normal development of the organism [15]. The intestine is the primary digestive and absorptive organ in aquaculture animals and is strongly correlated with growth [16]. Research has demonstrated the significant role of intestinal microbes in preserving host health and facilitating intestinal absorption [17, 18]. In this study, growth performance, oxidative capacity, intestinal health, and microflora of shrimp were used to evaluate the quality of partially replacing FM with ESBM. This study will provide guidance on the use of partial replacement of FM with ESBM in shrimp aquaculture.

## 2. Materials and Methods

### 2.1. Shrimp Maintenance and Diet Preparation

Healthy *Penaeus vannamei*, with an average body weight of 2.5 g, were procured from Ningde Shrimp Farm in Fujian, China. They were acclimated in a seawater-recirculating aquaculture system for 1 week before the experiment. We employ a rapid physical decapitation method to minimize the shrimp's pain. All shrimp handling procedures followed the guidelines outlined in the “Guide for the Care and Use of Laboratory Animals” of Fujian Agriculture and Forestry University and were approved by the university.

The proportion of FM in the basal feed of this experiment was 34%. ESBM was used to replace 5%, 10%, and 15% of FM in the FM group, respectively, to formulate four groups of equal nitrogen and equal fat test feeds (designated as FM, ESBM5, ESBM10, and ESBM15) (Table 1). The nutritional compositions of FM, ESBM5, ESBM10, and ESBM15 were shown in Table 2. All ingredients, except fish-soluble juice, squid paste, and standardized phospholipids, were finely pulverized and sieved through an 80-mesh sieve. The components were then accurately weighed according to the formulation proportions and blended in a mixer. During mixing, premeasured and well-mixed fish sauce, squid paste, standardized phospholipids, and 40% pure water were added. Sedimentary strips of 2 mm particle size were prepared using a bulking machine. The feed was dried and matured in an oven at 80°C for 30 min and then naturally dried indoors and stored at a low temperature (−20°C).

### 2.2. Feeding Management

The aquaculture experiment was carried out in the aquaculture workshop of Sheng Sheng Fisheries Technology Co. Ltd in Ningde. The shrimp fry was obtained from Zhangzhou Dabeinong Aquatic Co. Ltd and temporarily reared for 1 week. Robust and uniform-sized shrimp fry were randomly divided into four groups, with each group having three replicates, each containing 120 tails. The shrimp fry was fed three times a day (5:00, 11:00, and 17:00) at 3%–5% of the total body weight. Water changes were performed twice every morning and evening at a rate of 1/5, maintaining specific hydration indexes: water temperature (27–30°C), pH (7.8–8.3), salinity (5‰–8‰), dissolved oxygen (5.3–6.8 mg/L), and ammonia nitrogen (0.00–0.20 mg/L). Water quality monitoring is performed one time per day.

### 2.3. Sample Collection

At the end of the culture cycle (56 days), all shrimp were starved for 24 h before sampling. The total number and weight of shrimp in each bucket were recorded prior to sample collection to calculate weight gain rate (WGR), special growth rate (SGR), survival rate (SR), and feed conversion rate (FCR), with specific calculation formula as defined in previous studies [3].

Twenty shrimp were randomly selected from each bucket and dissected rapidly on ice packs. The hepatopancreas, muscle, and intestine were taken in cryopreservation tubes and stored in liquid nitrogen at −80°C for enzyme activity and gene expression analyses. Another 10 shrimp with intact intestinal tracts were preserved using RNA later for a separate intestinal microbiota analysis. Additionally, 10 shrimp with their intestinal contents removed from the midgut (2 cm) were preserved in Sevir's fixative for intestinal sectioning and histological observation.

### 2.4. Enzyme Activity Assay

For enzyme activity analysis, the shrimp hepatopancreas samples were mixed with precooled saline (4°C, pH 7.0) at a ratio of 1:9 (g:mL). Subsequently, homogenization was performed using a glass homogenizer at low temperature, followed by centrifugation at 4000 rpm and 4°C for 20 min. The enzyme activity was determined based on the collected supernatant. Amylase (C016-1-1), lipase (A054-2-1), trypsin (A080-2), total antioxidant capacity (A015-2-1), superoxide dismutase (A001-3), catalase (A007), and glutathione (A006) activities were assessed using the Nanjing Jiengcheng Institute of Bioengineering's assay kits (Nanjing Jiancheng Bioengineering Institute, Nanjing, China).

### 2.5. DNA Extraction

DNA from the intestine of three shrimp from each pond was extracted using the QIAamp PowerFecal DNA Kit (Qiagen, Germany) according to the manufacturer's instructions. Total genomic DNA was extracted using the PowerFecal DNA Isolation Kit (MoBio, Palo Alto, CA, USA) following the provided protocols. The concentration and quality of the DNA were assessed using a NanoVuePlus Spectrophotometer (GE Healthcare, USA) and 1% agarose gel electrophoresis. The V4 region of the 16S rRNA gene was amplified using two specific primers, as previously described [25]. After purification by QIAquick Gel Extraction Kit (Qiagen, Germany), PCR products were used to generate the sequencing libraries.

### 2.6. Intestinal Health and Microflora

For intestinal health analysis, the samples were subjected to intestinal sectioning and photographed at Xavier Biotechnology Co. Ltd. Nine tissue sections were prepared for each experimental group. ImageJ software was used to measure the villus height and villus width in each section 10 times. The DNA sequencing and analysis were completed by Beijing Nuohezhiyuan Biotechnology Co. Ltd (Beijing, China). Quantitative Insights Into Microbial Ecology (QIIME; http://qiime.org/index.html) was used to filter the raw data [26]. The unique and shared OTUs between the two groups were analyzed using the Draw Venn Diagram online tool (http://bioinformatics.psb.ugent.be/webtools/Venn/). Alpha diversity was analyzed using QIIME. The significance of differences in the alpha diversity index was assessed using the Wilcoxon test in R software (version 2.15.3). Beta diversity, including the unweighted pair group method with arithmetic means (UPGMA) tree and principal coordinates analysis (PCoA), was calculated using QIIME and weighted correlation network analysis (WGCNA), respectively [27].

### 2.7. Real-Time PCR

For all the collected samples, total RNA was extracted with RNeasy Mini Kit (Qiagen) and reverse transcribed into cDNA with PrimeScript II 1st Strand cDNA Synthesis Kit (Takara) according to a previous study [28]. Quantitative real-time PCR analysis was performed on a QuantStudio 5 (using the 96-well module) in a 10 μL volume containing 5 μL 2 × Polarsignal qPCR mix (MIKX.MKG800, China), 1 μL cDNA template, 0.5 μL forward primer, 0.5 μL backward primer, and 3 μL double-distilled water. PCR procedure of each assay in triplicate was used as follows: 40 cycles of amplification for 5 min at 95°C; 30 s at 95°C, 30 s at 60°C, and 15 s at 70°C. Data were normalized to the level of the *EF-1α* gene in each sample using the 2^−*ΔΔ*Ct^ method. All real-time PCR primers are listed in Table S1.

### 2.8. Statistical Analyses

All statistical analyses were performed using GraphPad Prism (GraphPad Software). Data are expressed as mean ± standard deviation. Prior to conducting a one-way ANOVA analysis, we verified the normality of the data using the Shapiro–Wilk test and assessed its homogeneity of variance through the Levene's test. Following these checks, we employed Duncan's test to compare the mean values across various treatments, with statistical significance set at *p* < 0.05. All experiments were performed three or more times independently.

## 3. Result

### 3.1. Growth Performance and Feed Utilization of Shrimp Fed the Experimental Diets

**Figure 1:**
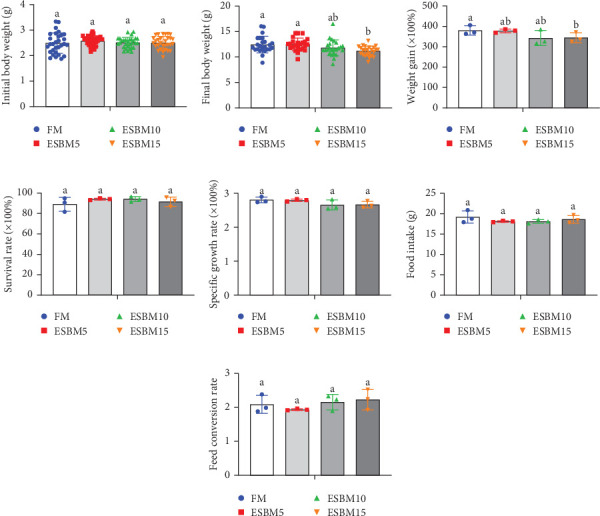
The effects of replacing FM with different ESBMs in feed on growth performance and feed utilization of shrimp *Penaeus vannamei*. (A) Initial body weight of shrimp fed the experimental diets (*n* = 30). (B) Final body weight of shrimp fed the experimental diets (*n* = 24). (C) Weight gain of shrimp fed the experimental diets (*n* = 3). (D) The survival rate of shrimp fed the experimental diets (*n* = 3). (E) The specific growth rate of shrimp fed the experimental diets. (F) Food intake of shrimp fed the experimental diets (*n* = 3). (G) The feed conversion rate of shrimp fed the experimental diets (*n* = 3). The dots indicating different treatment groups indicated data points in the graphs. Values with different letters indicated significant differences by one-way ANOVA, followed by Dunnett's post hoc test (*p* < 0.05).

Replacing FM with different ESBMs (5%–15%) in the shrimp diet did not result in significant differences in SR, specific growth rate, food intake rate, and food conversion rate compared to the control (Figure 1D–G). However, replacing FM with 15% ESBM in the shrimp diet reduces the final body weight and weight gain of shrimp (Figure 1A–C). These findings suggested that replacing FM with ESBM at 5%–10% levels in shrimp diets had no impact on shrimp growth performance and feed utilization, whereas the replacement of FM with 15% ESBM negatively affected shrimp growth.

### 3.2. Digestive Enzyme Activities and Oxidative Enzyme Activities of Shrimp Fed the Experimental Diets

**Figure 2:**
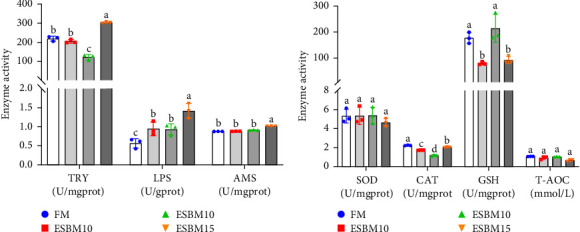
The effects of replacing FM with different ESBM in feed on digestive enzyme activities and oxidative capacity of shrimp *Penaeus vannamei*. (A) The digestive enzyme activity of shrimp fed the experimental diets (*n* = 3). (B) The oxidative capacity of shrimp fed the experimental diets. The dots indicating different treatment groups indicated data points in the graphs (*n* = 3). Values with different letters indicated significant differences by one-way ANOVA, followed by Dunnett's post hoc test (*p* < 0.05).

Experimental analysis of digestive enzyme activities showed that replacing FM with 5% ESBM in shrimp diets did not affect TRY and AMS but increased LPS enzyme activity compared to the control. Replacing FM with 10% ESBM did not affect AMS but inhibited TRY enzyme activity and increased LPS enzyme activity. Replacement of FM with 15% ESBM increased TRY, AMS, and LPS enzyme activities (Figure 2A).

Experimental analysis of oxidative enzyme activities revealed that replacement of FM with 5%–15% ESBM in shrimp diets had no effect on SOD and T-AOC but inhibited CAT enzyme activities compared to the control. Replacement of FM with 5% and 15% ESBM in shrimp diets inhibited GSH enzyme activity, but 10% replacement had no effect on GSH enzyme activity (Figure 2B).

### 3.3. The Expression Levels of Oxidative Stress-Related Genes and Growth Performance-Related Genes in Shrimp Fed the Experimental Diets

**Figure 3:**
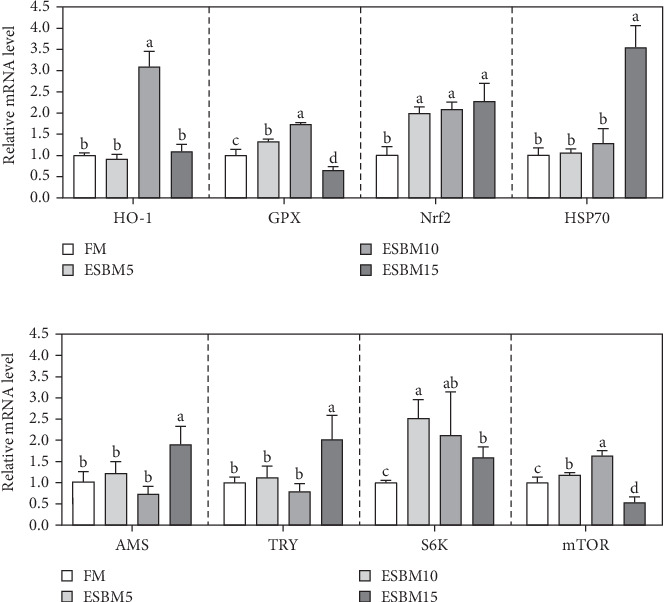
The effects of replacing FM with different ESBMs in feed on gene expression levels in shrimp *Penaeus vannamei*. (A) The expression levels of oxidative stress-related genes in the hepatopancreas of shrimp fed the experimental diets. (B) The expression levels of growth performance-related genes in the muscle of shrimp fed the experimental diets. Values with different letters indicated significant differences by one-way ANOVA, followed by Dunnett's post hoc test (*n* = 3, *p* < 0.05).

More and more studies have shown that HO-1, GPX, Nrf2, and HSP70 are crucial for oxidative stress resistance in shrimp [29–31]. The RT-qPCR analysis revealed that replacing FM with 5% ESBM in shrimp diets increased GPX and Nrf2 expression in hepatopancreas compared to the control, while it did not affect HO-1 and HSP70 expression. Additionally, replacing FM with 10% ESBM in shrimp diets increased HO-1, GPX, and Nrf2 expression, without affecting HSP70 expression. When FM was replaced with 15% ESBM in shrimp diets, the Nrf2 and HSP70 expression was increased, and GPX expression was inhibited but did not affect HO-1 expression (Figure 3A).

Several studies have confirmed that S6K, mTOR, AMS, and TRY are central to shrimp growth 31–34]. It was found that the expression levels of S6K and mTOR were upregulated in muscle compared with control after feeding shrimp diets with FM was replaced with 5% or 10% ESBM, which did not affect AMS and TRY expression. Furthermore, replacing FM with 15% ESBM in shrimp diets increased AMS, TRY, and S6K expression compared to the control but inhibited mTOR expression (Figure 3B).

### 3.4. The Effects of Intestinal Microflora in Shrimp Fed the Experimental Diets

**Figure 4:**
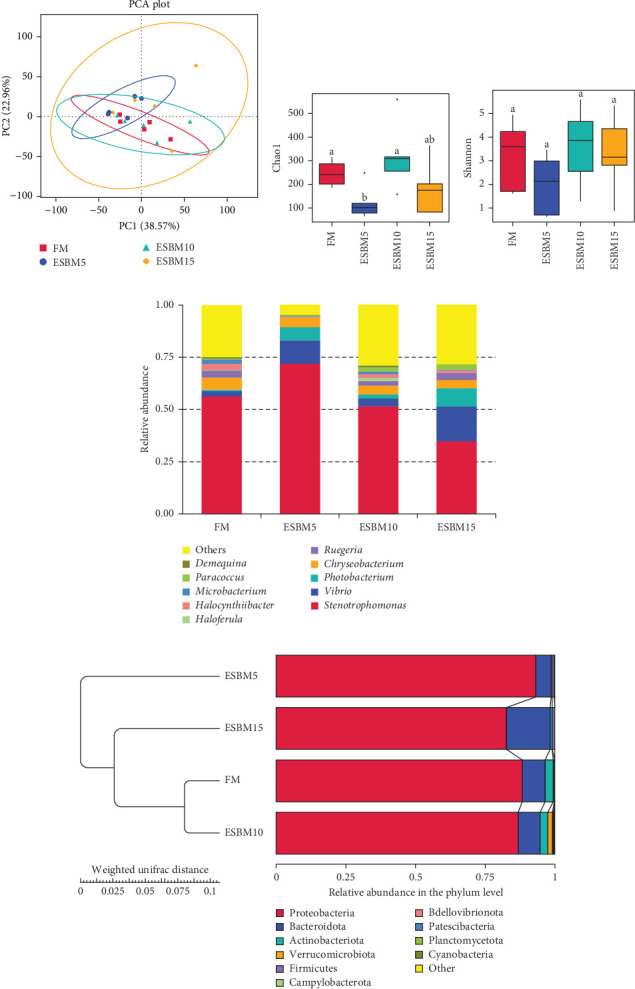
The effects of replacing FM with different ESBMs in feed on intestinal microflora in shrimp *Penaeus vannamei*. (A) The analysis of PCA in shrimp fed the experimental diets. (B and C) The α-diversity comparison including the Chao1 index and Shannon index in shrimp fed the experimental diets. Values with different letters indicated significant differences by one-way ANOVA, followed by Dunnett's post hoc test (*p* < 0.05). (D) Structure and composition of the bacterial communities in shrimp gut (*n* = 5) fed the experimental diets on the phylum level of taxonomy. (E) UPGMA clustering g based on all OTUs from the bacteria in the shrimp gut (*n* = 5) fed the experimental diets.

A principal coordinate analysis (PCA) revealed that the bacterial communities were different in shrimp fed the experimental diets (Figure 4A). The α-diversity in replacing FM with 5% and 15% ESBM groups was significantly lower than replacing FM with 0% and 10% ESBM groups, as supported by Chao1 index, and there was no difference between replacing FM with 0% ESBM group and 10% group (Figure 4B). Notably, the relative abundance of *Vibrio* was increased in replacing FM with 5% and 15% ESBM groups than replacing FM with 0% and 10% ESBM groups. Additionally, the relative abundance of *Microbacterium* was decreased in replacing FM with 5% and 15% ESBM groups than groups with 0% and 10% ESBM instead of FM (Figure 4D). UPGMA clustering further revealed the significant difference in bacterial communities in shrimp fed the experimental diets (Figure 4E). The group with 0% ESBM instead of FM was clustered on a branch with the group with 10% ESBM instead of FM (Figure 4E). The relative abundance of *Actinobacteria* phylum was similar in replacing FM with 0% or 10% ESBM groups, while it was reduced in group with 5% or 15% ESBM instead of FM compared to groups with 0% or 10% ESBM instead of FM (Figure 4E).

### 3.5. The Effects of Intestinal Health in Shrimp Fed the Experimental Diets

**Figure 5:**
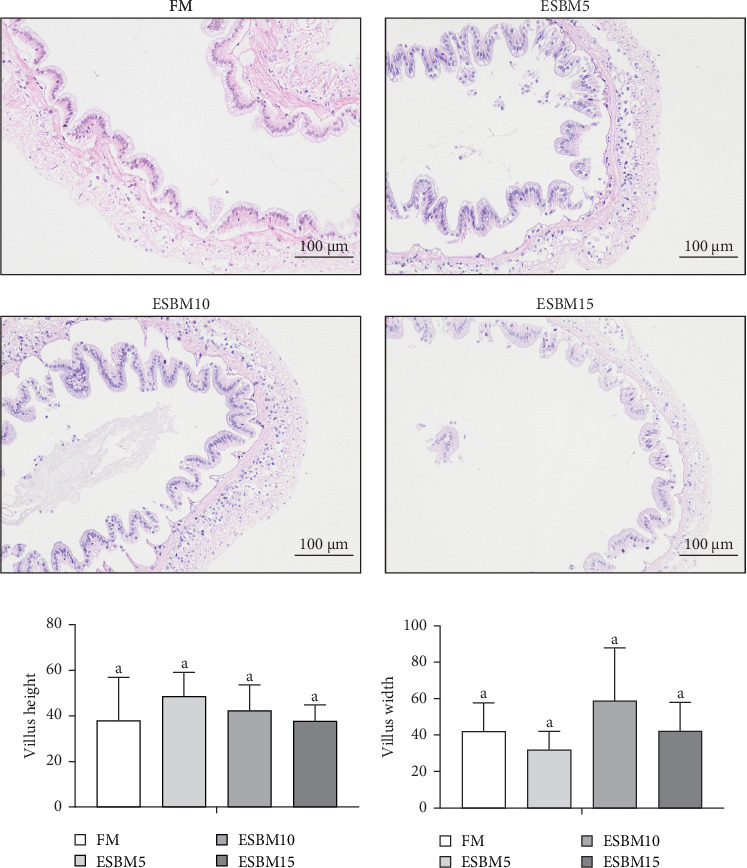
The effects of replacing FM with different ESBMs in feed on intestinal morphology in shrimp *Penaeus vannamei*. (A) Representative image of intestine histochemistry (stained by hematoxylin and eosin) in shrimp fed the experimental diets. The scale bar = 100 μm. (B) Villus height and (C) villus width in shrimp gut (*n* = 5) fed the experimental diets. Values with different letters indicated significant differences by one-way ANOVA, followed by Dunnett's post hoc test (*p* < 0.05).

The intestinal morphometry of shrimp *Penaeus vannamei* fed the experimental diets is shown in Figure 5A. The villus height and villus width in replacing FM with different ESBM groups were not different compared to the control (Figure 5B,C). Therefore, replacing FM with different ESBM has no effect on the intestinal health of shrimp.

## 4. Discussion

The search for alternative protein sources to substitute FM has been a prominent concern in aquaculture. Our recent results have already revealed that the partial replacement of FM with protein hydrolysates does not affect the growth performance of shrimp [33]. However, finding other protein sources to replace FM remains urgent. Herein, we reported a novel replacement ingredient, ESBM, which partially replaces FM and has no negative impact on shrimp growth performance. Specifically, replacing FM with 5% or 10% ESBM in the shrimp diet did not result in significant differences in growth performance and feed utilization.

In this study, replacing FM with 15% ESBM in the diet inhibited the growth of shrimp, indicating that excessive replacement of FM with ESBM is inappropriate (Figure 1B–C). This finding was supported by the inhibition of mTOR gene expression and the promotion of the harmful bacterium *Vibrio* in the intestine (Figures 3B and 4D). As a key intracellular mediator of nutrition sensing and energy regulation, the mTOR pathway regulates cell growth in response to stimuli such as nutrients, growth factors, energy, and environmental stress [35]. Some studies have revealed that the mTOR signaling pathway is closely related to host growth and development [10]. The mTOR signaling pathway plays a key role in protein synthesis and degradation [36]. For example, mTOR signaling pathway can affect the activities of digestive enzymes such as trypsin, chymolysin, lipase, and amylase at the translational level in fish [37]. The addition of 2.5% leucine to a 30% protein diet enhanced the expression of mTOR pathway genes in crabs, thereby stimulating protein synthesis in tissues and organs and ultimately promoting growth [38]. The inhibitory effect of replacing FM with 15% ESBM in the diet on shrimp growth may be due to the inhibition of the expression of mTOR, a key gene in the mTOR signaling pathway. High concentrations of ESBM have been shown to inhibit mTOR expression [10]. This effect may be attributed to the increased content of ANFs in the soy protein, which suppress mTOR expression. Additionally, higher levels of ESBM were correlated with a lower essential amino acid index (EAAI), a potential factor contributing to the inhibition of mTOR expression, which warrants further investigation. Moreover, the research showed that an elevated abundance of pathogenic bacteria (*Vibrio*) in the intestine negatively impacts the host's growth [39]. And *Vibrio* could activate host immunity, making energy unavailable for growth [40]. *Vibrio* is opportunistic pathogens of shrimp, which is known to be the major infectious agent of shrimp diseases, such as acute hepatopancreas necrosis [41]. Therefore, variation of the relative abundance of *Vibrio* has been used frequently as a parameter to estimate the risk of shrimp disease qualitatively [42]. *Vibrio* infection triggered the nuclear translocation of the shrimp transcription factor Dorsal, which regulated the expression of antimicrobial peptides [43]. *Vibrio* infection also induced the phagocytosis of shrimp hemocytes [44]. Another reason for the inhibitory effect of 15% ESBM in feed in place of FM on the growth of shrimp may be due to the promotion of the harmful bacteria *Vibrio* to colonize the intestines in shrimp, which needs further investigation. In addition, we found that the relative abundance of *Actinobacteria* phylum and *Microbacterium* was increased in replacing 0% and 10% ESBM instead of FM groups than FM with 5% and 15% ESBM group (Figure 4). Many *Actinomycetes* are known to function as probiotics, producing various nutrients that promote the growth and survival of farmed animals [45]. Additionally, they enhance the immunity of aquatic animals by enhancing the expression of immune indicators, thereby increasing their tolerance to external stressors [45]. Furthermore, *Actinomycetes* produce antagonistic compounds that exhibit antimicrobial, antiviral, antibiofilm, and antiquorum sensing activities [45]. Therefore, one reason for the replacing FM with 10% ESBM in the diet that did not affect the growth performance of shrimp *Penaeus vannamei* may be the higher content of probiotic *Actinomycetes* in the intestine, which need further confirmation. It is worth noting that partial replacement of FM with ESBM in the diet was able to inhibit the activity of CAT enzyme activity (Figure 2B). CAT enzyme is one of the key enzymes in the biological defense system [46]. Therefore, partial replacement of FM with ESBM in the diet may be able to affect the immunity of shrimp, showing some drawbacks.

Previous studies have demonstrated that HO-1, GPX, Nrf2, and HSP70 are crucial for oxidative stress resistance in shrimp [29–31]. It was found that the partial replacement of FM with ESBM in the diet promoted HO-1 and Nrf2 expression, suggesting that it may enhance shrimp's oxidative stress capacity, helping them adapt to environmental changes. It was noteworthy that GPX expression and CAT enzyme activity exhibited opposite results. This discrepancy may be attributed to negative feedback regulation of gene expression or functional differences between GPX and CAT, which require further investigation. Additionally, we observed that high concentrations of ESBM induced HSP70 expression, which has been linked to enhanced temperature adaptation [47], suggesting that ESBM may improve shrimp's ability to adapt to high temperatures, which needs further confirmation.

Although the replacement of FM with SBM has been extensively studied, the use of ESBM as a replacement for FM is still under-researched. Enzymatic processing of SBM enhances its protein content, increases small-molecule peptides, and eliminates various ANFs, making it more suitable for animal consumption [7]. The research showed that partial replacement of FM with ESBM in diets has been found not to affect or improved the growth performance of piglets, broilers, and abalone [7, 8, 10]. Replacing FM with 2% ESBM in the diet improved broilers' intestinal microbiota and growth performance [8, 9]. In aquaculture, replacing FM with 75% ESBM does not affect the growth performance and immunity of abalone [10]. Therefore, the proportion of different species suitable for replacing ESBM varies. The likely cause of this difference is the considerable variation in their diets and habits, which warrants further investigation. Our study revealed that replacing FM with 10% ESBM in the diet did not affect the growth performance of shrimp *Penaeus vannamei*, suggesting that partial replacement of FM with ESBM in diets has a promising application in aquaculture.

## 5. Conclusion

In conclusion, our study demonstrated that replacing FM with 10% ESBM did not affect the growth performance of shrimp. These findings not only enrich our knowledge of FM proteinogen replacement but also provide a reference for the use of ESBM as a substitute for FM in commercial feeds for shrimp *Penaeus vannamei* as well as other shrimp species.

## Figures and Tables

**Table 1:** Composition and proximate analysis of the experimental diets.

Ingredient (g/100 g)	FM	ESBM5	ESBM10	ESBM15
Fish meal^a^	34	32.3	30.6	28.9
Enzyme-treated soybean meal^a^	0	2.2	4.4	6.6
Soybean meal^a^	10	10	10	10
Chellocken meal^a^	10	10	10	10
Krill meal^a^	7.5	7.5	7.5	7.5
Wheat flour^a^	27.5	26.9	26.3	25.7
Fish oil	2	2.1	2.2	2.3
Squid meal^a^	3	3	3	3
Soy lecithin	1	1	1	1
Brewer's yeast	2	2	2	2
Vitamin premix^b^	1.5	1.5	1.5	1.5
Mineral premix^b^	1.5	1.5	1.5	1.5
Proximate analysis	—	—	—	—
Moisture	9.24	9.34	9.55	9.42
Crude protein	43.14	43.17	42.89	42.96
Crude lipid	8.65	8.74	8.39	8.52
Ash	11.73	11.32	11.28	10.89

^a^These materials were supplied by Haima Feed Corporation, Fuzhou, China.

^b^Vitamin premix and mineral premix with specific component, as defined in previous studies [19].

**Table 2:** The essential amino acid profile (%) of experimental diets.

Amino acid	FM	ESBM5	ESBM10	ESBM15	Requirement for shrimps
Arginine	2.60	2.58	2.57	2.55	1.90 [20]
Histidine	1.46	1.44	1.42	1.39	0.80 [21]
Isoleucine	1.85	1.83	1.82	1.80	1.00 [21]
Leucine	3.06	3.04	3.01	2.98	1.70 [21]
Lysine	3.11	3.06	3.00	2.96	2.10 [20]
Methionine	1.12	1.08	1.06	1.03	0.90 [22]
Phenylalanine	1.81	1.80	1.79	1.78	1.40 [21]
Threonine	1.73	1.71	1.69	1.67	1.40 [23]
Valine	2.23	2.20	2.18	2.15	1.40 [24]

## Data Availability

The data that support the findings of this study are available from the corresponding author upon request.

## Supporting Information

Supporting Information Table S1. All real-time PCR primers are listed in Table S1.
