# Supplementary material for: Enzyme-Treated Soybean Meal Serves as an Effective Alternative to Fishmeal in the Diet of the Shrimp Penaeus vannamei
Source: Aquac Nutr. 2025 Mar 24;2025:2312302. doi: 10.1155/anu/2312302 (PMC11957856; doi:10.1155/anu/2312302)
Supplement: Supporting Information — Table S1. All real-time PCR primers are listed in Table S1. [file 2312302.f1.docx]

Supplemental Table 1 Sequences of primer used in this paper

| Name | Sequence（5'-3'） | Comments |
| --- | --- | --- |
| Q-HO-1-F | GCATGGCAGTGACCGAGATTGA | qPCR |
| Q-HO-1-R | GTCGCTGCTTCGTCTCCTCATC |  |
| Q-GPX-F | AGGGACTTCCACCAGATG | qPCR |
| Q-GPX-R | CAACAACTCCCCTTCGGTA |  |
| Q-Nrf2-F | GATGAGAAGCGAGCCAGAGCG | qPCR |
| Q-Nrf2-R | GCCGTCGGATGTCTCGGATAA |  |
| Q-HSP70-F | GCGTACTGCCTGTGAGCG | qPCR |
| Q-HSP70-R | CGGGTGATGGAGGTGTAGAAA |  |
| Q-AMS-F | CTCTGGTAGTGCTGTTGGCT | qPCR |
| Q-AMS-R | TGTCTTACGTGGGACTGGAAG |  |
| Q-TRY-F | CGGAGAGCTGCCTTACCAG | qPCR |
| Q-TRY-R | TCGGGGTTGTTCATGTCCTC |  |
| Q-S6K-F | GCAAGAGGAAGACGCCATA | qPCR |
| Q-S6K-R | CCGCCCTTGCCCAAAACCT |  |
| Q-mTOR-F | TGCCAACGGGTGGTAGA | qPCR |
| Q-mTOR-R | GGGTGTTTGTGGACGGA |  |
| Q-EF-1α-F | GTATTGGAACAGTGCCCGTG | qPCR |
| Q-EF-1α-F | ACCAGGGACAGCCTCAGTAAG |  |
